# Knockdown of *HSPA9* induces TP53-dependent apoptosis in human hematopoietic progenitor cells

**DOI:** 10.1371/journal.pone.0170470

**Published:** 2017-02-08

**Authors:** Tuoen Liu, Kilannin Krysiak, Cara Lunn Shirai, Sanghyun Kim, Jin Shao, Matthew Ndonwi, Matthew J. Walter

**Affiliations:** 1 Department of Medicine, Division of Oncology, Washington University School of Medicine, St. Louis, Missouri, United States of America; 2 Siteman Cancer Center, Washington University School of Medicine, St. Louis, Missouri, United States of America; Cincinnati Children's Hospital Medical Center, UNITED STATES

## Abstract

Myelodysplastic syndromes (MDS) are the most common adult myeloid blood cancers in the US. Patients have increased apoptosis in their bone marrow cells leading to low peripheral blood counts. The full complement of gene mutations that contribute to increased apoptosis in MDS remains unknown. Up to 25% of MDS patients harbor and acquired interstitial deletion on the long arm of chromosome 5 [del(5q)], creating haploinsufficiency for a large set of genes including *HSPA9*. Knockdown of *HSPA9* in primary human CD34+ hematopoietic progenitor cells significantly inhibits growth and increases apoptosis. We show here that HSPA9 knockdown is associated with increased TP53 expression and activity, resulting in increased expression of target genes *BAX* and *p21*. HSPA9 protein interacts with TP53 in CD34+ cells and knockdown of HSPA9 increases nuclear TP53 levels, providing a possible mechanism for regulation of TP53 by HSPA9 haploinsufficiency in hematopoietic cells. Concurrent knockdown of TP53 and HSPA9 rescued the increased apoptosis observed in CD34+ cells following knockdown of HSPA9. Reduction of HSPA9 below 50% results in severe inhibition of cell growth, suggesting that del(5q) cells may be preferentially sensitive to further reductions of HSPA9 below 50%, thus providing a genetic vulnerability to del(5q) cells. Treatment of bone marrow cells with MKT-077, an HSPA9 inhibitor, induced apoptosis in a higher percentage of cells from MDS patients with del(5q) compared to non-del(5q) MDS patients and normal donor cells. Collectively, these findings indicate that reduced levels of HSPA9 may contribute to TP53 activation and increased apoptosis observed in del(5q)-associated MDS.

## Introduction

Myelodysplastic syndromes (MDS) are a heterogeneous group of blood cancers characterized by abnormal hematopoietic cell maturation, increased apoptosis of bone marrow cells, and peripheral blood cytopenias. Low blood counts result in morbidity and mortality due to bleeding and infection. A fraction of patients will progress from MDS to secondary acute myeloid leukemia (AML), characterized by a reduction in apoptosis, the etiology of which is incompletely understood. Up to 25% of MDS patients acquire an interstitial deletion on chromosome 5q, also known as del(5q). Two distinct commonly deleted regions (CDR) on the long arm of chromosome 5 have been identified in patients with MDS and AML. The distal CDR spans chromosome 5q33.1 (148.6–151.1 Mb) and contains 44 genes, including *RPS14*.[1–3] The proximal CDR spans chromosome 5q31.2 (136.3–138.6 Mb) and contains 30 genes including *HSPA9*, and is associated with patients that progress from MDS to secondary AML, or have therapy–related MDS and AML.[2–4] Our group, and others, hypothesize that haploinsufficiency of genes on del(5q) contribute to increased apoptosis and peripheral blood cytopenias observed in MDS.

The human *HSPA9* gene is located on chromosome 5q31.2 and encodes for one of the heat shock protein 70 family members, also known as mortalin/mthsp70/PBP74/GRP75. HSPA9 is localized in the mitochondria, endoplasmic reticulum, plasma membrane, cytoplasmic vesicles and cytosol.[5] As a molecular chaperone, HSPA9 interacts with other proteins and functions in regulating cellular stress response, cell proliferation and apoptosis.[6] The role of HSPA9 in hematopoiesis has been studied in multiple models. Knockdown of *HSPA9* in primary human CD34+ hematopoietic progenitors inhibits erythroid cell maturation and increases apoptosis.[7] Zebrafish with a homozygous point mutation in *Hspa9* present phenotypically with a variety of abnormalities including severe anemia, defects in erythroid differentiation and elevated apoptosis.[8] Knockdown of *Hspa9* in a mouse bone marrow transduction transplantation model showed reduction in hematopoietic stem and progenitor cells [9,10] and heterozygous deletion of *Hspa9* in mice alters B-cell lymphopoiesis.[11] Collectively, these studies revealed important functions of HSPA9 in hematopoiesis, suggesting it may contribute to the increased apoptosis observed in MDS.

Increased apoptosis in del(5q) MDS cells is associated with elevated levels of TP53 in erythroid cells.[12,13] There is evidence that haploinsufficiency of genes on del(5q) contribute to increased TP53 and apoptosis. Reduced levels of *RPS14*, a 5q33.1 gene, increases apoptosis of human progenitor cells via increased TP53 levels.[12] We hypothesize that multiple genes on del(5q) may contribute to apoptosis through TP53 activation. Prior studies in non-hematopoietic cells show HSPA9 binds to the cytoplasmic sequestration domain of TP53 and knockdown of HSPA9 results in relocation of TP53 from the cytoplasm to the nucleus.[5,14–16] It is not known whether reduced expression of HSPA9 in primary human hematopoietic cells results in activation of TP53 and the induction of apoptosis. In this study, we examine the relationship between the levels of HSPA9 and TP53 in human CD34+ hematopoietic progenitor cells, elucidating their potential interaction and regulation of apoptosis in del(5q)-associated MDS.

## Methods

### Cells and reagents

Granulocyte colony-stimulating factor (G-CSF) mobilized peripheral blood hematopoietic progenitor cells (CD34+) were obtained from Fred Hutchinson Cancer Research Center (Seattle, WA, USA) (purity of CD34+ cells were higher than 90%). MDS bone marrow samples were collected on a protocol approved by the Human Research Protection Office at Washington University. The study was carried out in accordance with the Declaration of Helsinki and amendments and informed written consent was obtained from all subjects prior to enrollment. MKT-077 was obtained from Sigma-Aldrich (St Louis, MO, USA).

### Lentiviral shRNA vectors and production

The production of pLK0.1 lentiviral shRNAs has been described previously.[10] shRNA sequences are listed in S1 Table.

### Lentiviral shRNA vector transduction and culture

Transduction of lentiviral shRNA (S1 Table) into human CD34+ cells and associated culture conditions have been described previously.[10] Briefly, CD34+ progenitor cells were primed in X-VIVO 15 medium (Lonza, Walkersville) containing human cytokines (50 ng/mL of stem-cell factor [SCF], 50 ng/mL of Fms-related tyrosine kinase 3 ligand [FLT3-ligand], 50 ng/mL of thrombopoietin [TPO], and 50 ng/mL of interleukin 3 [IL-3] [PeproTech]) with L-glutamine (Thermo Fisher Scientific) overnight before lentiviral transduction. Cells were spinoculated in the presence of polybrene (5 μg/mL) and incubated overnight at 37°C in 5% CO_2_. Cells were washed and incubated in erythroid differentiation media containing serum-free expansion medium (StemCell Technologies) with 25 ng/mL of SCF, 10 ng/mL of IL3, 10 ng/mL of IL6, 0.5 units of erythropoietin (EPO), 100 U/mL of penicillin/streptomycin (P/S), and L-glutamine for 7 days. All cytokines were obtained from Peprotech.

### Western blotting

Cell lysates were prepared in radioimmunoprecipitation assay (RIPA) buffer as described previously.[10] The following antibodies were used: TP53 (SC-126; Santa Cruz Biotechnology), Bax (554104; BD Pharmagen), HSPA9 (MA3-028; Affinity BioReagents), β-actin (A5441; Sigma-Aldrich), and lamin B1 (ab16048; Abcam). Densitometry was performed using ImageJ (http://imagej.nih.gov/ij/) (S2 Table).

### Cytosol and nuclear fraction isolation

The nuclear and cytoplasmic fractions of CD34+ cells were isolated using a previously published protocol.[17] 1x10^7^ CD34+ cells were harvested by centrifuging at 500g for 5 minutes and then washed with PBS. The cell pellet was resuspended with 1 ml of ice-cold HLB buffer (10 mM Tris [pH 7.5], 10 mM NaCl, 3 mM MgCl2, 0.3% [vol/vol] NP-40 and 10% [vol/vol] glycerol) and incubated on ice for 10 minutes. The cell suspension was then centrifuged at 800g for 8 minutes at 4°C. The supernatant contains the cytosol fraction. The nuclear pellet was washed four times by HLB buffer and centrifuged at 200g for 2 minutes at 4°C. The nuclear pellet was then resuspended in 0.5 ml ice-cold NLB (20 mM Tris [pH 7.5], 150 mM KCl, 3 mM MgCl2, 0.3% [vol/vol] NP-40 and 10% [vol/vol] glycerol). The nuclei were sonicated three times at 20% power for 15 seconds in an ice bath with 2 minutes of cooling between each sonication. The sonicated nuclei were then centrifuged at 16,000g for 15 minutes at 4°C and the supernatant was collected. The cytosol and nuclear fractions were used for Western blotting. Beta-actin and lamin B were used to measure the purity of cytosol and nuclear fractions, respectively.

### RT-PCR

Cells were harvested by centrifugation at 1500 g for 5 minutes at 4°C, resuspended in 250 μL 1X PBS, then lysed by adding 750 μL Trizol LS reagent (Invitrogen). RNA was then isolated following the manufacturer’s protocol. RNA was resuspended in 30 μL of RNase-free water and the RNA concentration was measured using a NanoDrop 2000 spectrophotometer (ThermoFisher scientific, Wilmington, DE, USA). The TURBO DNA-free kit (Thermo Fisher Scientific) was used to remove DNA contamination in the RNA samples. The first-strand cDNA was synthesized using the SuperScript III first-strand synthesis system. FAM-MGB primer/probe mixes for *TP53* (Hs01034249_m1), *HSPA9* (Hs00945576_g1), *GAPDH* (Hs02758991_g1), *BAX* (Hs00180269_m1) and *p21* (Hs00355782_m1) were used for RT-PCR TaqMan gene expression assays (Applied Biosystems). All RT-PCR reactions were performed in duplicate with no-RT control and water control on a StepOne Plus Real time PCR System (Applied Biosystems). Individual cDNA samples were normalized according to their levels of *GAPDH* and the relative standard curve method was used for analysis.

### Luciferase reporter assay

CD34+ cells were transduced with shGFP, sh433 (shHSPA9 #2) and sh960 (shHSPA9 #1) and cultured for 5 days as previously reported.[10] PG13 and renilla plasmids (a gift from Dr. Zaika) were electroporated into the CD34+ cells using Amaxa human CD34+ cell nucleofector (program U-008) following the manufacturer’s protocol (Lonza, Allendale, NJ).[18] Two days after electroporation, luciferase and renilla activity were measured using the Renilla luciferase assay system from Promega (Madison, WI) following manufacturer’s protocol. Plates were read using AD/LD analysis software from Beckman Coulter LD40 (Pasadena, CA).

### Flow cytometry

Flow cytometry was used to measure the apoptotic cells using Annexin V as previously described.[10] Intracellular flow cytometry to measure TP53 has been described previously.[12] The following antibodies were used: anti-CD71 (FITC, 11-0719-42, ebioscience), anti-glycophorin A (PE, 12-9987-80, ebioscience), anti-TP53 (Alexa647, #2533S, cell signaling) and Annexin V (556421, BD Pharmingen).

### Gene set enrichment analysis

CD34^+^/CD71^-^ cells were isolated using Fluorescence-activated cell sorting (FACS) from 4 independent cultures of CD34+ cells differentiated in erythroid cytokines for 5 days following transduction with sh433 (shHSPA9 #2) or shLUC. We isolated RNA as described above and performed Affymetrix Gene 1.0 ST mRNA arrays. Gene Set Enrichment Analysis (GSEA) was performed using a set of well-annotated TP53-target genes and p21-inhibited genes.[19] The Affymetrix data has been deposited in GEO (GSE78164).

### Immunoprecipitation

The immunoprecipitation method has been described previously.[20] Briefly, 2x10^7^ cells were harvested after 5 days in erythroid culture and lysed in 1 ml 1X RIPA buffer on ice for 30 minutes with sonication. The lysates were centrifuged at 16,000g for 10 minutes and the supernatant was collected and used in the immunoprecipitation assay. 50 μL of TP53 antibody-conjugated agarose beads (sc-126 AC, Santa Cruz) or mouse IgG−agarose beads (A0919, Sigma) were washed three times with 1X RIPA buffer followed by centrifugation at 100g for 3 minutes at 4°C and the supernatant was discarded. The lysates were then incubated with the TP53 antibody-conjugated agarose beads or mouse IgG−agarose beads for 1 hour at room temperature with rotation. After incubation, the mixture was centrifuged at 100g for 3 minutes at 4°C and the supernatant was used for Western blotting analysis. The immunoprecipitate was washed three times with 0.9% saline. After washing, the immunoprecipitated mixture was eluted with 200 μL of 0.1M glycine. All fractions at different stages were collected and used for Western blotting assay.

### Immunofluorescence and fluorescence digital imaging microscopy

Cells were washed 2X with PBS, and transferred by cytospin to Superfrost Plus Microscope Slides (Fisher). The cells were then fixed with 0.4% paraformaldehyde (diluted in PBS) for 20 minutes at room temperature. The cells were washed 3X with PBS, and permeabilized with 0.3% Triton X-100 (diluted in PBS) at 4°C overnight. The cells were then washed 3X with 0.1% Tween 20 in PBS (PBST), and incubated in 10% donkey serum in PBST (blocking buffer) for 1 hour. After blocking, cells were incubated with monoclonal TP53 antibody (DO-1, Santa Cruz Biotechnology; diluted 1:200 in blocking buffer) overnight. The cells were washed 3X with PBST, and secondary staining was performed by incubating with Alexa Fluor594-conjugated goat polyclonal antibody to mouse IgG (ab150120, Abcam; diluted 1:200 in blocking buffer) for 2 hours at room temperature. The cells were then washed 3X with PBST. Mounting reagent, Prolong Gold Antifade Reagent with DAPI (P36931, Invitrogen) was then applied onto the cell surface, covered with a coverslip, and sealed. Fluorescent imaging was then performed through a 40X objective, using a Zeiss LSM 700 microscope.

### Statistics

Statistical significance of the data between two groups was analyzed by the Student t-test (Prism 5). Statistical significance of the data with more than two groups was analyzed by one-way ANOVA with a Tukey post-test (Prism 5). Significance levels were set at p<0.05 (labelled *), p<0.01 (labelled **), and p<0.001 (labelled ***).

## Results

### Knockdown of HSPA9 increases TP53 levels and activity in primary human CD34+ progenitor cells

**Fig 1:**
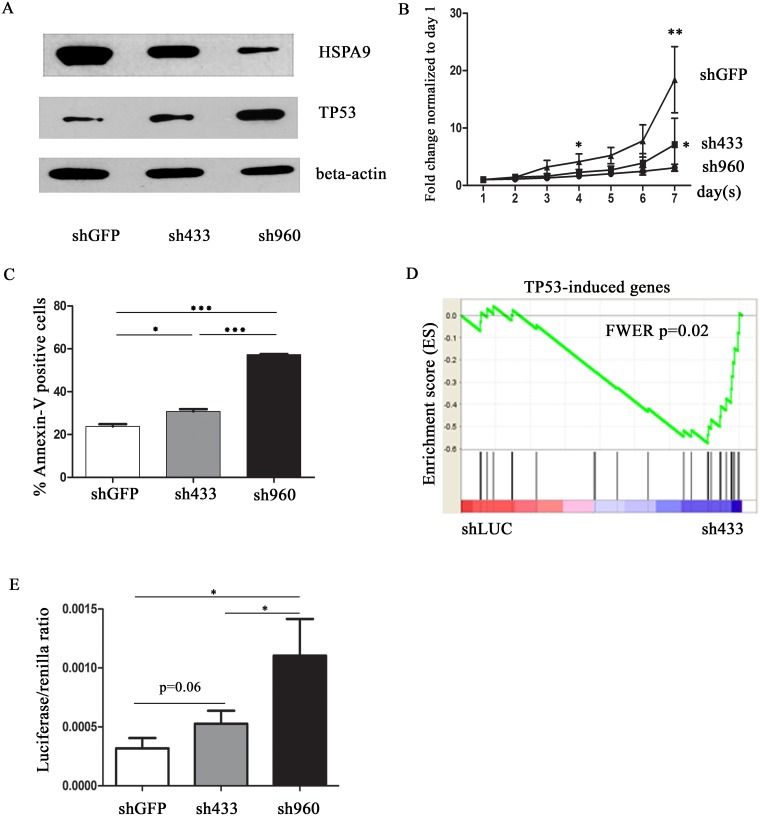
Knockdown of HSPA9 increases TP53 expression and activity in CD34+ cells. (A) Representative immunoblot of HSPA9 and TP53 protein in CD34+ cells transduced with shRNAs targeting a control gene (GFP) or HSPA9 after 7 days in erythroid culture conditions. (B) Transduced cells were seeded at equal concentrations in erythroid culture media and the total number of cells was counted daily in the presence of puromycin selection. The fold change in cell counts was calculated relative to the number of cells on day 1 (n = 3 independent replicates for each shRNA). (C) Quantification of Annexin V+ cells (a surrogate for apoptosis) following knockdown of HSPA9 in CD34+ cells (n = 3 for each shRNA). (D) Gene Set Enrichment Analysis (GSEA) enrichment score plot for 26 annotated TP53-induced genes. Individual genes in the gene set are represented by a black vertical bar in the middle of the plot. (E) TP53 luciferase reporter assay showed a dose-dependent increase in TP53 activity following HSPA9 knockdown (n = 3). LUC, luciferase. * p<0.05, ** p<0.01, *** p<0.001.

We used primary human CD34+ hematopoietic progenitor cells grown in unilineage erythroid expansion media to explore the mechanism of increased apoptosis observed in erythroid cells from del(5q) MDS patients. In order to test the effects of HSPA9 knockdown in human CD34+ hematopoietic progenitor cells, we utilized two previously reported short hairpin RNAs targeting the human *HSPA9* gene (sh433 and sh960).[10] sh433 and sh960 previously reduced the HSPA9 protein level to approximately 50% and 20%,[10] respectively (and in Fig 1A), and mRNA level to 64%±4% (p<0.01) and 39% ± 8% (p<0.01), respectively, compared to control shGFP (S1A Fig). Consistent with previous results, knockdown of HSPA9 inhibits cell growth and increased apoptosis of CD34+ cells cultured in erythroid media (Fig 1B and 1C).[10] To identify potential genes contributing to this phenotype, we flow sorted CD34^+^/CD71^-^ cells from 4 independent cultures, 5 days after transduction with shLUC (control) and sh433 (shHSPA9 #2 in prior study).[10] sh433 was chosen to approximate haploinsufficient levels of *HSPA9* mRNA observed in MDS samples with del(5q) compared to non-del(5q) samples.[4] We isolated CD34^+^/CD71^-^ cells in order to study progenitor cells. We chose day 5 because CD34^+^/CD71^-^ cells had similar cell cycle and apoptotic profiles at that time (data not shown), allowing us to identify dysregulated genes that precede changes in cell cycle and levels of apoptosis, and not as a consequence of these processes. We isolated RNA and performed Affymetrix Gene 1.0 ST mRNA arrays. *HSPA9* mRNA was reduced by ~50% in the knockdown samples on the arrays (expression level of sh433 versus shLUC = 1684 ± 18.24 versus 3615 ± 125.8 [arbitrary units], respectively, p<0.001, N = 4). Unsupervised clustering analysis did not consistently segregate the samples, indicating that massive transcriptional changes did not occur at this time point, as expected based on the experimental design. However, using supervised approaches (Gene Set Enrichment Analysis [GSEA]), we identified significantly altered genes in HSPA9 knockdown cells, including upregulation of TP53-induced genes (shLUC versus sh433, FWER = 0.02) (Fig 1D and S3 Table).[19,21]

We used several methods to explore the relationship between HSPA9 and TP53 levels. We observed that *TP53* mRNA levels were increased following knockdown of *HSPA9* after 7 days in erythroid culture conditions (S1B Fig). We also confirmed an increase in TP53 protein levels in sh433 and sh960 transduced cells by Western blotting (Fig 1A). Finally, we used intracellular flow cytometry to determine whether TP53 induction was restricted to specific subsets of CD34+ derived erythroid cells. We observed that sh960 significantly enhanced intracellular TP53 levels compared to shGFP, regardless of CD71 expression (p<0.05) (S2 Fig). These results suggest that knockdown of HSPA9 increases *TP53* transcription and translation. Next, we used a luciferase reporter assay to measure the functional activity of TP53. Four days after transduction of CD34+ cells with shRNAs (using shGFP as a control), TP53 luciferase reporter and renilla plasmids were co-electroporated into cells and the luciferase and renilla activity were measured 48 hours after electroporation. The luciferase/renilla ratio was significantly higher in sh960 transduced cells compared to shGFP control (sh960 versus shGFP, p<0.05; sh433 versus sh960, p = 0.06, Fig 1E). Collectively, the data indicate that knockdown of HSPA9 increases TP53 activity in CD34+ cells.

### Knockdown of HSPA9 increases the expression of TP53 target genes p21 and BAX in CD34+ cells

**Fig 2:**
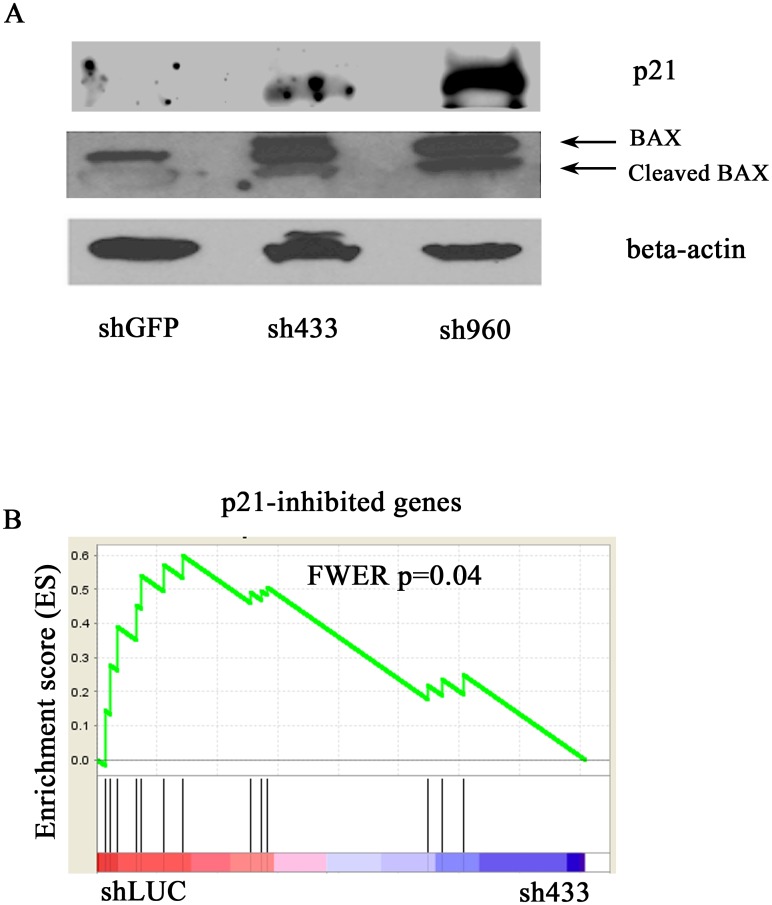
Knockdown of HSPA9 increases expression of the TP53 target genes p21 and BAX in CD34+ cells. (A) Representative immunoblot of p21 and BAX following knockdown of HSPA9 in CD34+ cells. (B) Gene Set Enrichment Analysis (GSEA) enrichment score plot for 13 annotated p21-inhibited genes. Individual genes in the gene set are represented by a black vertical bar in the middle of the plot.

*p21* (*CDKN1A*), a cell cycle regulator, and *BAX*, a pro-apoptotic gene, were two of the highest expressed TP53-target genes in our erythroid culture gene chip analysis, suggesting that they may contribute to the observed phenotype. We confirmed an increase in p21 and BAX protein (Fig 2A) and mRNA levels (S1C and S1D Fig respectively) in HSPA9 knockdown cells. Known p21-repressed genes were also significantly decreased in HSPA9 knockdown cells (shGFP versus sh433, FWER p = 0.04) (Fig 2B), further implicating TP53 and p21 as potential mediators of the cell cycle changes previously observed in HSPA9 knockdown cells.[10]

### HSPA9 interacts with TP53 in CD34+ cells and HSPA9 knockdown increases nuclear TP53 levels

**Fig 3:**
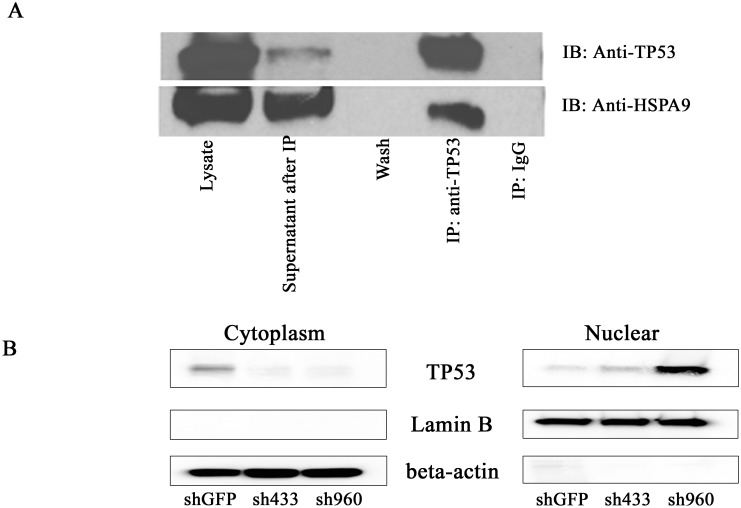
HSPA9 and TP53 interact in CD34+ cells and HSPA9 knockdown increases nuclear TP53 levels. (A) Representative immunoblots of TP53 and HSPA9 following immunoprecipitation with anti-TP53 or an IgG control. (B) Representative immunoblots of TP53 from the cytoplasmic and nuclear fractions of cell lysates following knockdown of HSPA9 in CD34+ cells grown in erythroid culture conditions for 5 days.

In order to further elucidate the relationship between TP53 and HSPA9, we performed immunoprecipitation (IP) of TP53 in untransduced CD34+ cells. We observed that HSPA9 is detected in the eluate following immunoprecipitation with a TP53 antibody. The IgG immunoprecipitation was used as the negative control, and neither protein was detected in this immunoprecipated control complex (Fig 3A). These results suggest a direct or indirect interaction between HSPA9 and TP53 in CD34+ cells. To further study the mechanism of knockdown of HSPA9 in regulating TP53 expression and activity, we isolated the cytosolic and nuclear fractions after knockdown of HSPA9 in CD34+ cells. Western blotting showed that knockdown of HSPA9 decreased TP53 levels in the cytoplasm and increased TP53 levels in the nucleus (Fig 3B), suggesting knockdown of HSPA9 leads to an increase in TP53 nuclear localization in CD34+ cells. The increase in nuclear TP53 following HSPA9 knockdown was also observed by immunofluorescence (S3 Fig**)**

### Reduction of TP53 inhibits the apoptosis induced by HSPA9 knockdown

**Fig 4:**
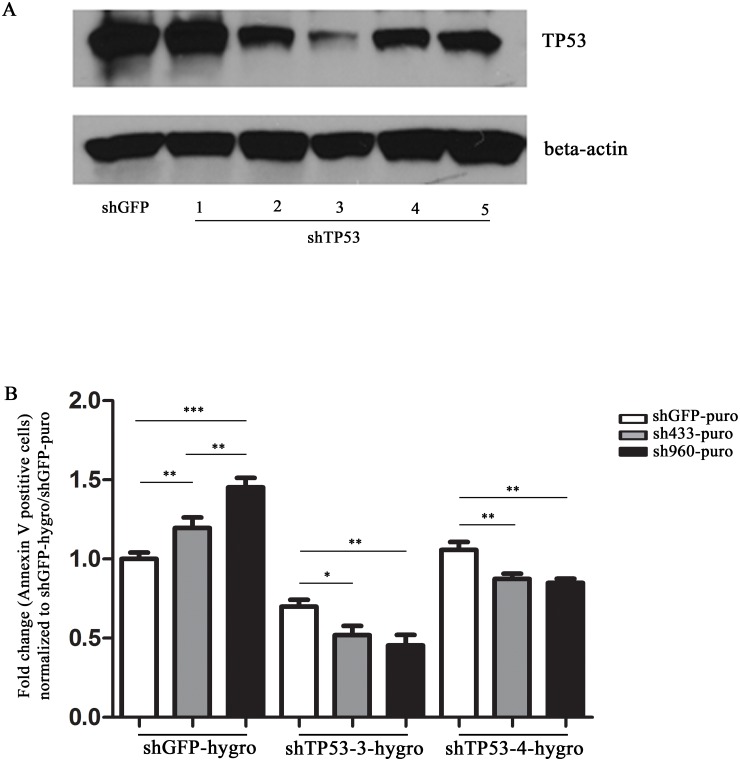
Reduction of TP53 inhibits apoptosis induced by HSPA9 knockdown. (A) Immunoblot of TP53 following knockdown of TP53 by shRNAs in CD34+ cells grown in erythroid culture conditions for 4 days. (B) CD34+ cells grown in erythroid culture conditions were co-transduced with lentiviral constructs carrying an shRNA targeting TP53 with a hygromycin resistance gene (e.g., shGFP, shTP53-3, or shTP53-4) and an shRNA targeting HSPA9 with a puromycin resistance gene (shGFP, sh433, or sh960). Cells were grown in the presence of both hygromycin and puromycin and the fold change in the percentage of Annexin V+ cells was measured by flow cytometry and normalized to the shGFP-hygromycin/shGFP-puromycin transduced cells (n = 3 technical replicates, representative of 2 independent experiments). *p<0.05, **p<0.01,***p<0.001.

We have shown that knockdown of HSPA9 can increase TP53 levels and induce apoptosis. Next, we asked whether reducing the levels of TP53 could inhibit the apoptosis induced by knockdown of HSPA9 in CD34+ cells. We tested 5 shRNAs targeting TP53 using a lentiviral construct containing a hygromycin resistance gene. After comparing knockdown achieved by each of the 5 constructs, we proceeded with shTP53#3 and shTP53#4 which were able to decrease TP53 levels by about 80% and 50%, respectively, compared to control shGFP (Fig 4A). We next co-transduced lentiviruses containing shRNA targeting HSPA9 and TP53 into CD34+ cells, using double antibiotic selection with puromycin and hygromycin, respectively. Consistent with shRNAs targeting HSPA9 alone, cells transduced with shGFP-hygromycin and sh433-puromycin or sh960-puromycin increased the percentage of Annexin V positive cells compared to shGFP-puromycin. The apoptosis following HSPA9 knockdown compared to shGFP was significantly decreased following simultaneous knockdown of TP53 with shTP53#3 and shTP53 #4 (p<0.05) (Fig 4B, S4 Fig).

### Pharmacologic inhibition of HSPA9 induces apoptosis in CD34+ cells

**Fig 5:**
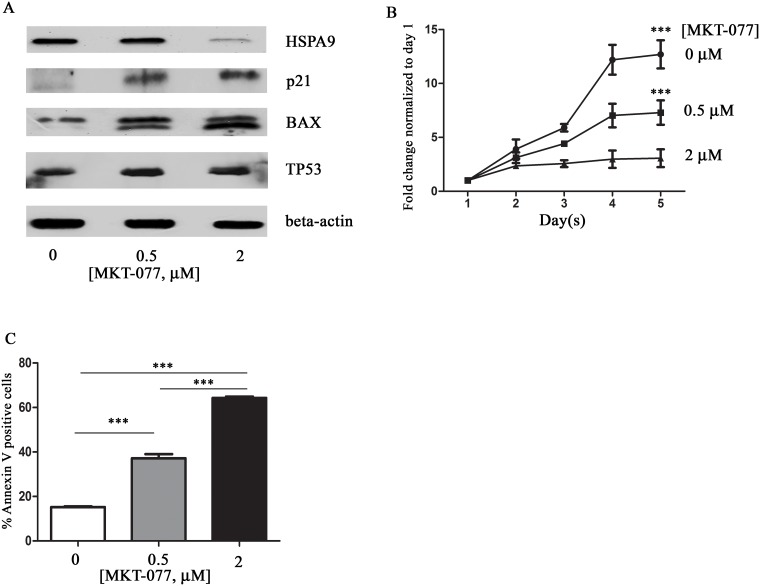
HSPA9 inhibitor MKT-077 reduces cell growth, induces apoptosis, and increases p21 and BAX expression in CD34+ cells. (A) Immunoblot of HSPA9, p21, BAX and TP53 following MKT-077 treatment of CD34+ cells grown in erythroid culture conditions for 5 days. (B) CD34+ cells were seeded at equal concentrations in erythroid culture conditions, and the total number of cells was counted daily in the presence of MKT-077. The fold change in cell counts was calculated relative to the number of cells on day 1 (n = 3) (0.5 μM versus control, p<0.001; 2 μM versus control, p<0.001). (C) Quantification of Annexin V+ cells (a surrogate for apoptosis) following treatment of CD34+ cells with various concentrations of MKT-077 for 5 days (n = 3). ***p<0.001.

Next, we studied the effects of pharmacologic inhibition of HSPA9 on cell growth, apoptosis, and TP53 levels in CD34+ cells using MKT-077, an inhibitor of HSP70 protein family members including HSPA9.[22] Following 5 days of MKT-077 treatment of CD34+ cells, HSPA9 expression was reduced in a dose-dependent manner (Fig 5A). MKT-077 also inhibits CD34+ cell growth in a dose-dependent manner (Fig 5B) and increased apoptosis by day 5 (Fig 5C). TP53 protein levels (S5 Fig) and the expression of the TP53 target genes p21 and BAX were also increased following treatment with MKT-077 (Fig 5A).

### Cells with reduced HSPA9 are sensitive to MKT-077 treatment

**Fig 6:**
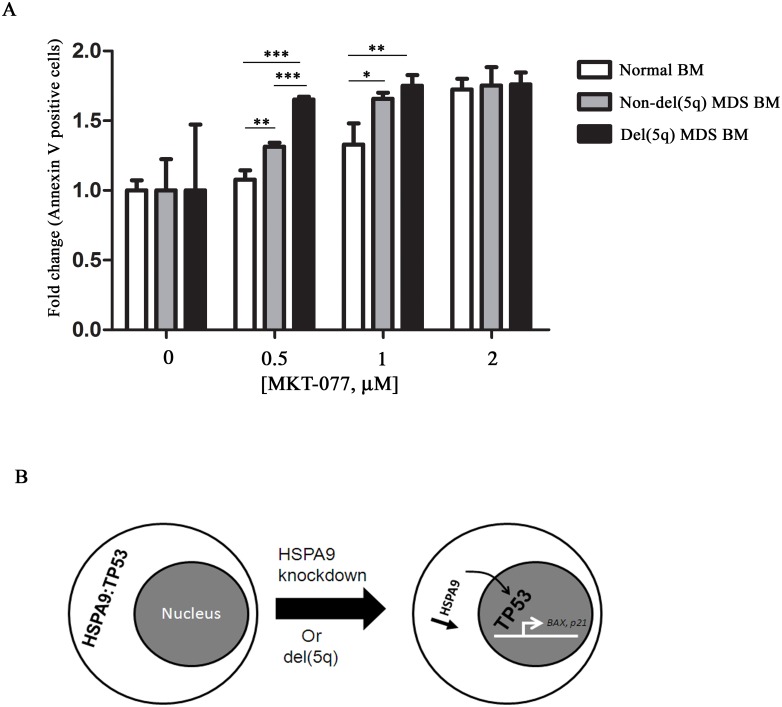
Del(5q)-associated MDS bone marrow cells are sensitive to MKT-077 treatment. (A) Bone marrow (BM) cells from a healthy donor (normal BM) and MDS patients without and with del(5q) (n = 1 each) were treated with various concentrations of MKT-077 for 4 days. The fold change in the percentage of Annexin V+ cells was measured by flow cytometry and normalized to 0 μM of MKT-077 (n = 3, technical replicates). (B) Model of the mechanism of TP53 activation following HSPA9 knockdown. HSPA9 interacts with TP53 in the cytoplasm. HSPA9 reduction leads to the release of TP53 from the HSPA9-TP53 cytoplasmic complex. Translocation of TP53 into the nucleus activates downstream target genes *BAX* and *p21*. *p<0.05, **p<0.01, ***p<0.001.

Haploinsufficiency of some genes can sensitize cells to further reductions in their level of expression. This concept has been proposed as a therapeutic approach to kill cancer cells harboring genes that are sensitive to complete loss of expression, referred to as CYCLOPS genes.[23] *HSPA9* is reduced by 50% in del(5q)-associated MDS cells, consistent with haploinsufficient levels.[4] We tested whether MDS samples with del(5q) spanning the HSPA9 gene maybe more sensitive to further HSPA9 reduction than MDS samples without del(5q) using MKT-077. While MKT-077 induced apoptosis in all BM cells in a dose-dependent manner, we observed that 0.5 μM of MKT-077 induced a higher percentage of apoptosis in del(5q)-associated MDS bone marrow cells (i.e., cells with reduced *HSPA9*) compared to non-del(5q) MDS cells and normal cells [non-del(5q) versus normal, p<0.01; del(5q) versus non-del(5q), p<0.001; del(5q) versus normal, p<0.01; Fig 6A, S4B–S4D Fig]. The increased apoptosis induced by MKT-077 in del(5q) MDS cells was associated with reduced HSPA9 levels compared to vehicle-treated cells (S6 Fig).

## Discussion

The present study addresses the effects of HSPA9 expression levels on apoptosis in hematopoietic cells. Del(5q) MDS bone marrow samples display increased apoptosis associated with an induction of TP53 in erythroid cells and upregulation of TP53 target gene expression.[12,13] Two commonly deleted regions (CDRs) exist at chromosome 5q33.1 (distal) and 5q31.2 (proximal) in patients with del(5q)-associated myeloid diseases. In this report, we observed that knockdown of HSPA9, a gene on the proximal chromosome 5 CDR, increased TP53 levels and functional activity, resulting in apoptosis in primary human hematopoietic progenitor cells. Knockdown of HSPA9 resulted in a dose-dependent increase in TP53 levels and transactivation of down-stream target genes, including pro-apoptotic BAX, and subsequently increased apoptosis. Apoptosis in knockdown cells was rescued by reducing TP53 levels, supporting that apoptosis induced by HSPA9 knockdown is TP53-dependent. Our data also suggest that reduction of HSPA9 below haploinsufficient levels is poorly tolerated by cells, potentially providing a genetic vulnerability that could be leveraged by further reducing HSPA9 levels in del(5q) cells. We show that del(5q)-associated MDS cells tend to be more sensitive to pharmacologic inhibition of HSPA9 compared to non-del(5q) cells. Collectively, the data suggest that haploinsufficiency of HSPA9 on del(5q) induces TP53 and may contribute to the accelerated apoptosis seen in del(5q)-associated MDS.

Haploinsufficiency of *RPS14*, a gene on the distal chromosome 5 CDR, has been shown to induce apoptosis in primary hematopoietic cells. Haploinsufficiency of *RPS14* results in increased TP53 levels in primary human erythroid cells and bone marrow cells from mice lacking one copy of *Rps14* alone [24], or in combination with 7 other contiguous genes.[12,25] Genetic deletion of *Tp53* in mice haploinsufficient for *Rps14* reversed many of the hematopoietic deficits induced by *Rps14* deletion, suggesting that elevated TP53 levels are responsible for many of the phenotypic abnormalities induced by *RPS14* haploinsufficiency.[24,25] How del(5q) hematopoietic cells gain clonal dominance in MDS when haploinsufficiency of several genes (e.g., *RPS14* and *HSPA9*) cause increased apoptosis is an active area of research. There is evidence that haploinsufficiency of the casein kinase 1A1 gene *(Csnk1a1)* in mice, located on the chromosome 5 distal CDR in humans, induces hematopoietic stem cell expansion and a competitive repopulation advantage.[26] Therefore, some del(5q) genes may induce apoptosis while others contribute to clonal dominance. In addition, many groups have shown that mutations or deletions of *TP53* are significantly associated with loss of chromosome 5 or del(5q) in myeloid malignancies.[27–31] It may be that mutation, deletion, or modulation of *TP53* is required for low-risk MDS cells with increased apoptosis to progress to high-risk MDS or secondary AML. Indeed, TP53 mutant cells can be found at low levels in patients with low-risk del(5q)-associated MDS and mutations are associated with evolution to acute myeloid leukemia.[32] Collectively, the data suggest that the cooperation of multiple del(5q) genes, and genes on other chromosomes (e.g., *TP53*), cooperate to induce the phenotypes observed in patients with MDS.

HSPA9 is a chaperone for many proteins. We show that HSPA9 and TP53 interact directly or indirectly in primary human hematopoietic cells, similar to that observed in non-hematopoietic cells.[15] Knockdown of HSPA9 leads to an increase in TP53 in the nucleus, resulting in transactivation of TP53 target genes and increased apoptosis (Fig 6B). Increased apoptosis in hematopoietic cells has been observed in various HSPA9 knockdown models, including primary human and mouse hematopoietic cells.[10,16] The known function of HSPA9 to protect cells from a variety of stresses may be partially mediated through its regulation of TP53, and haploinsufficiency may ultimately limit a cell’s ability to appropriately regulate stress states, including MDS. The level of HSPA9 expression and timing of deletion may be important for these phenotypes, as we have previously reported that constitutive heterozygous deletion of *Hspa9* in mice does not result in cytopenias.[11] This suggests that compensation for a constitutive deletion of Hspa9 may occur during mouse development. HSPA1A, another Hsp70 family member, has been shown to regulate erythropoiesis by preventing active caspase-3 from cleaving GATA1 in the nucleus and inducing apoptosis.[33] Expression of a nucleus-targeted HSPA1A in MDS samples rescued erythroid cell differentiation, implicating this Hsp70 family member in MDS pathogenesis.[34]

The activation of wild-type TP53 and induction of apoptosis by haploinsufficiency of del(5q) genes, including *RPS14* and *HSPA9*, may contribute to bone marrow failure and ineffective hematopoiesis in MDS.[35] Haploinsufficiency of genes may also create a genetic vulnerability that could be used to selectively kill del(5q)-containing cells. While we observed that ~50% reduction of HSPA9 increases apoptosis and reduces cell growth, >50% knockdown of HSPA9 severely limits hematopoietic cell growth *in vitro* and *in vivo*.[9,10] In addition, complete loss of Hspa9 in mice is early embryonic lethal, suggesting that reducing HSPA9 levels below 50% may be lethal to cells.[11] Consistent with complete loss of Hspa9 being lethal, HSPA9 has recently been identified as an essential gene in human cells.[36,37] Inherited variants in HSPA9 have also been associated with congenital sideroblastic anemia with data suggesting >50% reduction is required to cause this phenotype.[35] In cancer, the paradigm of modulating gene dosage as a therapeutic approach was experimentally established several years ago with the identification of CYCLOPS (copy number alterations yielding cancer liabilities owing to partial loss) genes.[23] To test whether reduction in HSPA9 below haploinsufficient levels cells could further increase apoptosis, we used MKT-077, a rhodacyanine dye that inhibits the activity of HSP70 family members, to treat HSPA9 knockdown cells and del(5q)-associated MDS samples.[22,38,39] While MKT-077 likely has a broad range of targets, we showed using this proof-of-concept approach that cells with reduced HSPA9 tended to be more sensitive to apoptosis induction following MKT-077 exposure compared to control cells. The successful paradigm of targeting a CYCLOPS gene on del(5q) has been elegantly shown to be the mechanism underlying the sensitivity of del(5q) cells to lenalidomide treatment.[23] Del(5q) cells are more sensitive to lenalidomide than normal cells because the drug induces degradation of CSNK1A1, a gene that is expressed at haploinsufficient levels in del(5q) cells compared to normal cells.[40]

The simultaneous loss of multiple genes on del(5q) likely contributes to the complex phenotypes observed in MDS.[26,35,41–49] Some genes contribute to increased apoptosis, thrombocytopenia, clonal dominance, B cell defects, and genetic susceptibility to treatments. The co-occurrence of TP53 mutations suggests a specific advantage, and possibly dependence, of these two genetic mutations in MDS cells. Future studies designed to eradicate TP53 mutant cells is a high priority to improve outcomes in patients with MDS.

## Supporting information

S1 Fig mRNA expression in CD34+ cells following HSPA9 knockdown. (A) Fold change in HSPA9 mRNA expression in CD34+ cells grown in erythroid culture conditions normalized to shGFP control cells (n = 3). (B) Fold change in TP53 mRNA expression in CD34+ cells grown in erythroid culture conditions normalized to shGFP control cells (n = 3). (C) Fold change in p21 mRNA expression in CD34+ cells grown in erythroid culture conditions normalized to shGFP control cells (n = 3). (D) Fold change in BAX mRNA expression in CD34+ cells grown in erythroid culture conditions normalized to shGFP control cells (n = 3).
(TIF)

S2 Fig Measurement of TP53 levels in CD34+ cells following knockdown of HSPA9. (A) (left panel) Representative histogram of intracellular TP53 levels measured by flow cytometry in bulk CD34+ cells grown in erythroid culture conditions following knockdown of HSPA9. (right panel) Mean fluorescence intensity (MFI) of TP53 following knockdown of HSPA9 (n = 3). (B) MFI of TP53 in CD71+ or CD71- cells following knockdown of HSPA9 in CD34+ cells, grown in erythroid culture conditions (n = 3). *p<0.05.
(TIF)

S3 Fig Immunofluorescence of TP53 in CD34+ cells following knockdown of HSPA9. Representative images of CD34+ cells transduced with lentiviral shRNA and cultured for 5 days. Antibody control represents CD34+ cells transduced with sh960 targeting HSPA9 and processed only with the secondary antibody, but not the primary antibody.
(TIF)

S4 Fig Measurement of apoptosis in cells transduced by various shRNAs or treated with MKT-077. (A) Non-normalized data presented in Fig 4B. CD34+ cells grown in erythroid culture conditions were co-transduced with lentiviral constructs carrying an shRNA targeting TP53 with a hygromycin resistance gene (e.g., shGFP, shTP53-3, or shTP53-4) and an shRNA targeting HSPA9 with a puromycin resistance gene (shGFP, sh433, or sh960). Cells were grown in the presence of both hygromycin and puromycin and the fold change in the percentage of Annexin V+ cells was measured by flow cytometry (n = 3 technical replicates). (B). Non-normalized data presented in Fig 6A. Bone marrow (BM) cells from a healthy donor (normal BM) and MDS patients without and with del(5q) (n = 1 each) were treated with various concentrations of MKT-077 for 4 days. The percentage of Annexin V+ cells was measured by flow cytometry (n = 3, technical replicates). (C) Bone marrow (BM) cells from a healthy donor (normal BM) and MDS patients without and with del(5q) (n = 1 each) were treated with various concentrations of MKT-077 for 4 days (non-overlapping samples with Fig 6A). The percentage of Annexin V+ cells was measured by flow cytometry (n = 3, technical replicates). (D) Non-normalized data presented above in panel C. The percentage of Annexin V+ cells was measured by flow cytometry (n = 3, technical replicates). *p<0.05, **p<0.01, ***p<0.001.
(TIF)

S5 Fig MKT-077 treatment increases TP53 levels in CD34+ cells following knockdown of HSPA9. (A) Mean fluorescence intensity (MFI) of TP53 following treatment with various doses of MKT-077 (n = 3 technical replicates, representative of 2 independent experiments). ***p<0.001.
(TIF)

S6 Fig HSPA9 levels are reduced in MDS cells following treatment with MKT-077. Bone marrow (BM) cells from a MDS patient with del(5q) were treated with various concentrations of MKT-077 for 4 days. Immunoblot of HSPA9 and beta-actin protein is shown.
(TIF)

S1 Table Short hairpin RNA sequences. (DOCX)

S2 Table Quantification of Western blot images by densitometry. (DOCX)

S3 Table TP53-induced and p21-inhibited gene lists used for GSEA analysis. (DOCX)
