# Supplementary figures and images for: Knockdown of HSPA9 induces TP53-dependent apoptosis in human hematopoietic progenitor cells
Source: PLoS One. 2017 Feb 8;12(2):e0170470. doi: 10.1371/journal.pone.0170470 (PMC5298293; doi:10.1371/journal.pone.0170470)

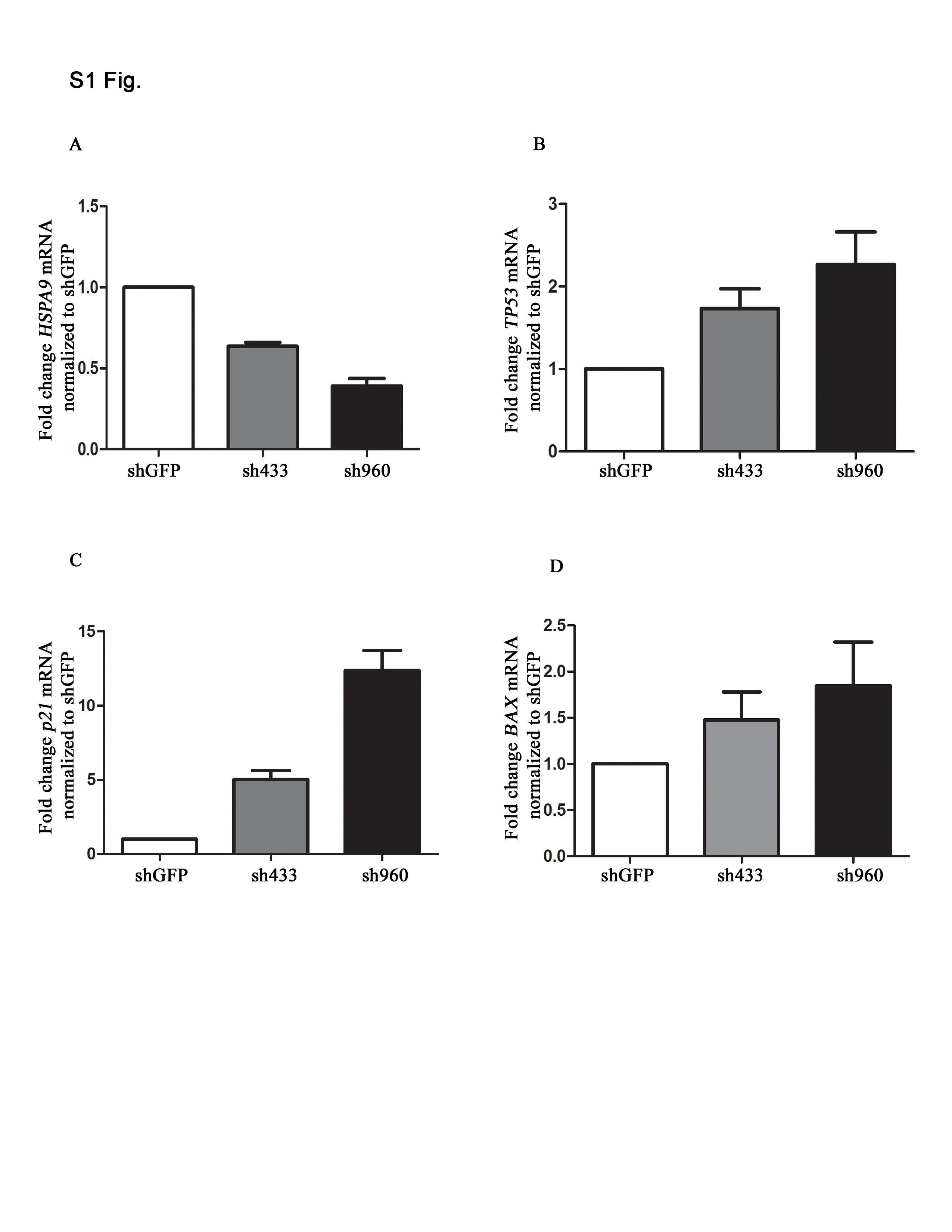

Supplement: S1 Fig — (A) Fold change in HSPA9 mRNA expression in CD34+ cells grown in erythroid culture conditions normalized to shGFP control cells (n = 3). (B) Fold change in TP53 mRNA expression in CD34+ cells grown in erythroid culture conditions normalized to shGFP control cells (n = 3). (C) Fold change in p21 mRNA expression in CD34+ cells grown in erythroid culture conditions normalized to shGFP control cells (n = 3). (D) Fold change in BAX mRNA expression in CD34+ cells grown in erythroid culture conditions normalized to shGFP control cells (n = 3). (TIF) [file pone.0170470.s001.tif]

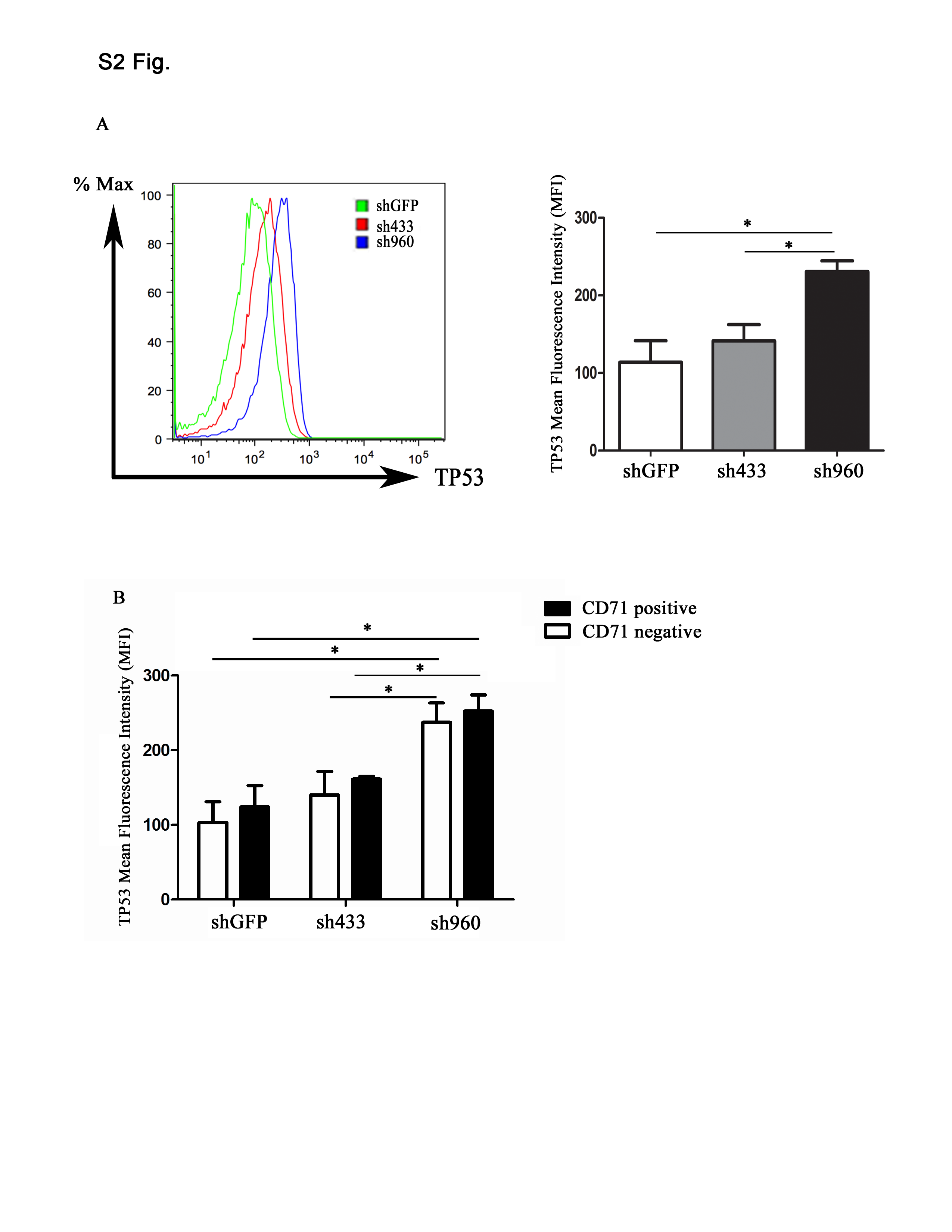

Supplement: S2 Fig — (A) (left panel) Representative histogram of intracellular TP53 levels measured by flow cytometry in bulk CD34+ cells grown in erythroid culture conditions following knockdown of HSPA9. (right panel) Mean fluorescence intensity (MFI) of TP53 following knockdown of HSPA9 (n = 3). (B) MFI of TP53 in CD71+ or CD71- cells following knockdown of HSPA9 in CD34+ cells, grown in erythroid culture conditions (n = 3). *p<0.05. (TIF) [file pone.0170470.s002.tif]

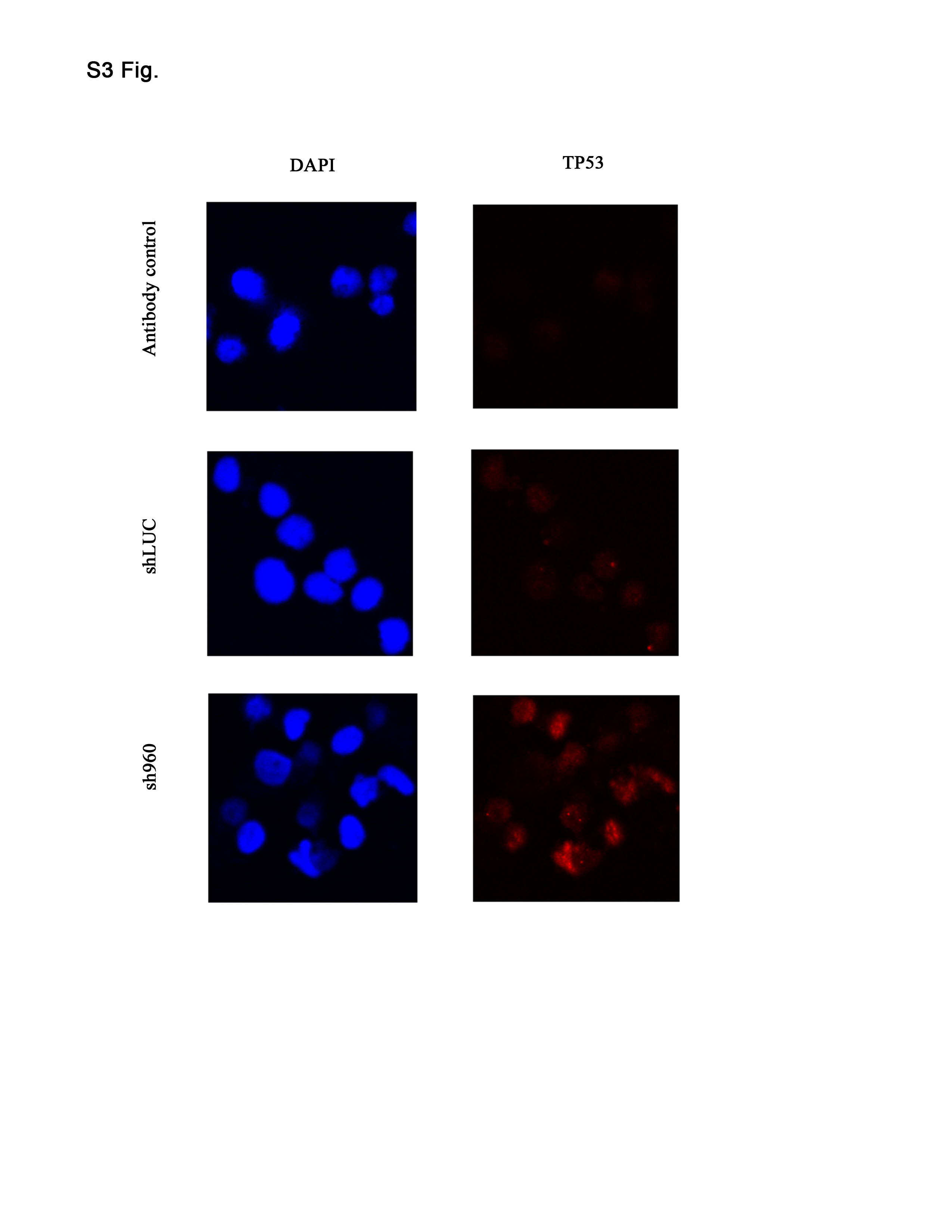

Supplement: S3 Fig — Representative images of CD34+ cells transduced with lentiviral shRNA and cultured for 5 days. Antibody control represents CD34+ cells transduced with sh960 targeting HSPA9 and processed only with the secondary antibody, but not the primary antibody. (TIF) [file pone.0170470.s003.tif]

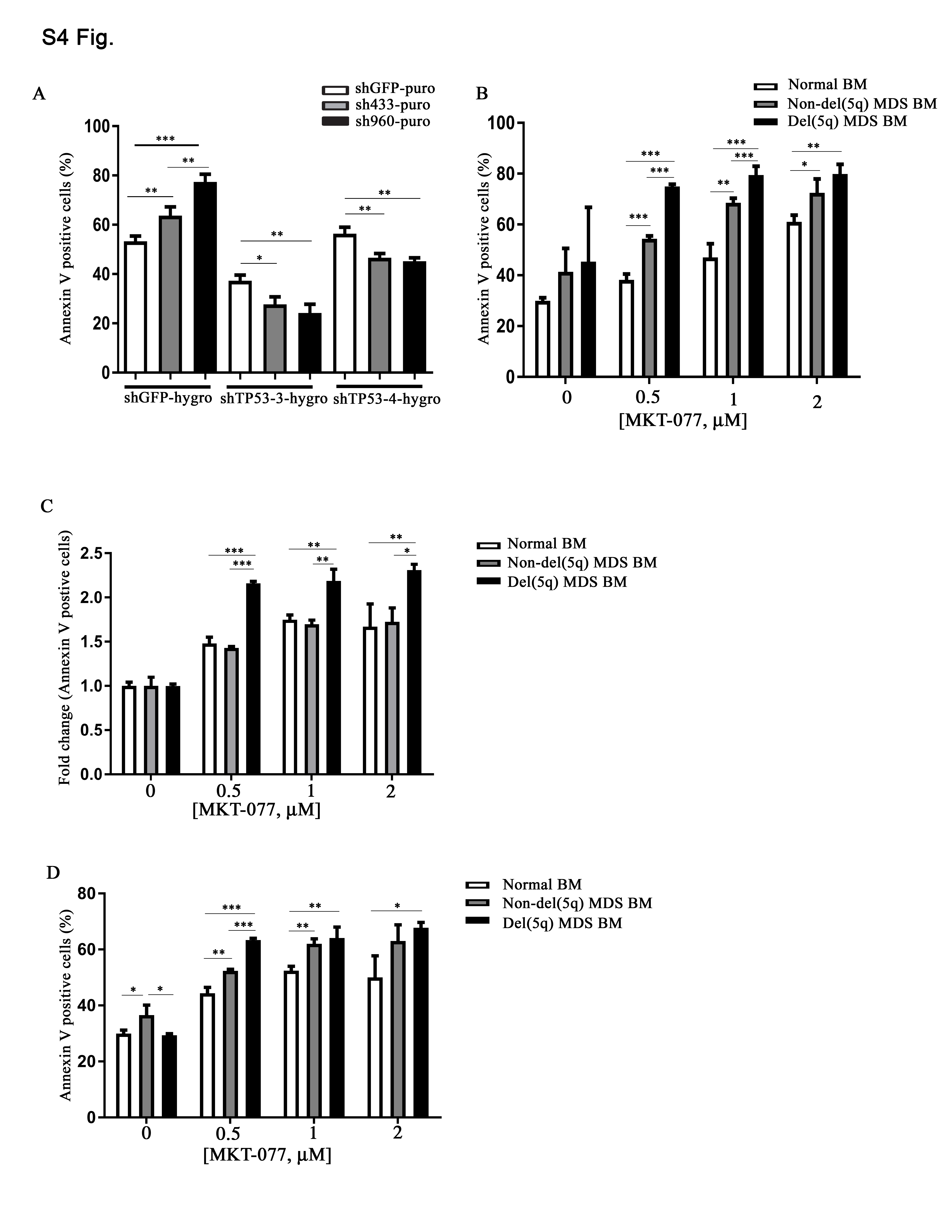

Supplement: S4 Fig — (A) Non-normalized data presented in Fig 4B. CD34+ cells grown in erythroid culture conditions were co-transduced with lentiviral constructs carrying an shRNA targeting TP53 with a hygromycin resistance gene (e.g., shGFP, shTP53-3, or shTP53-4) and an shRNA targeting HSPA9 with a puromycin resistance gene (shGFP, sh433, or sh960). Cells were grown in the presence of both hygromycin and puromycin and the fold change in the percentage of Annexin V+ cells was measured by flow cytometry (n = 3 technical replicates). (B). Non-normalized data presented in Fig 6A. Bone marrow (BM) cells from a healthy donor (normal BM) and MDS patients without and with del(5q) (n = 1 each) were treated with various concentrations of MKT-077 for 4 days. The percentage of Annexin V+ cells was measured by flow cytometry (n = 3, technical replicates). (C) Bone marrow (BM) cells from a healthy donor (normal BM) and MDS patients without and with del(5q) (n = 1 each) were treated with various concentrations of MKT-077 for 4 days (non-overlapping samples with Fig 6A). The percentage of Annexin V+ cells was measured by flow cytometry (n = 3, technical replicates). (D) Non-normalized data presented above in panel C. The percentage of Annexin V+ cells was measured by flow cytometry (n = 3, technical replicates). *p<0.05, **p<0.01, ***p<0.001. (TIF) [file pone.0170470.s004.tif]

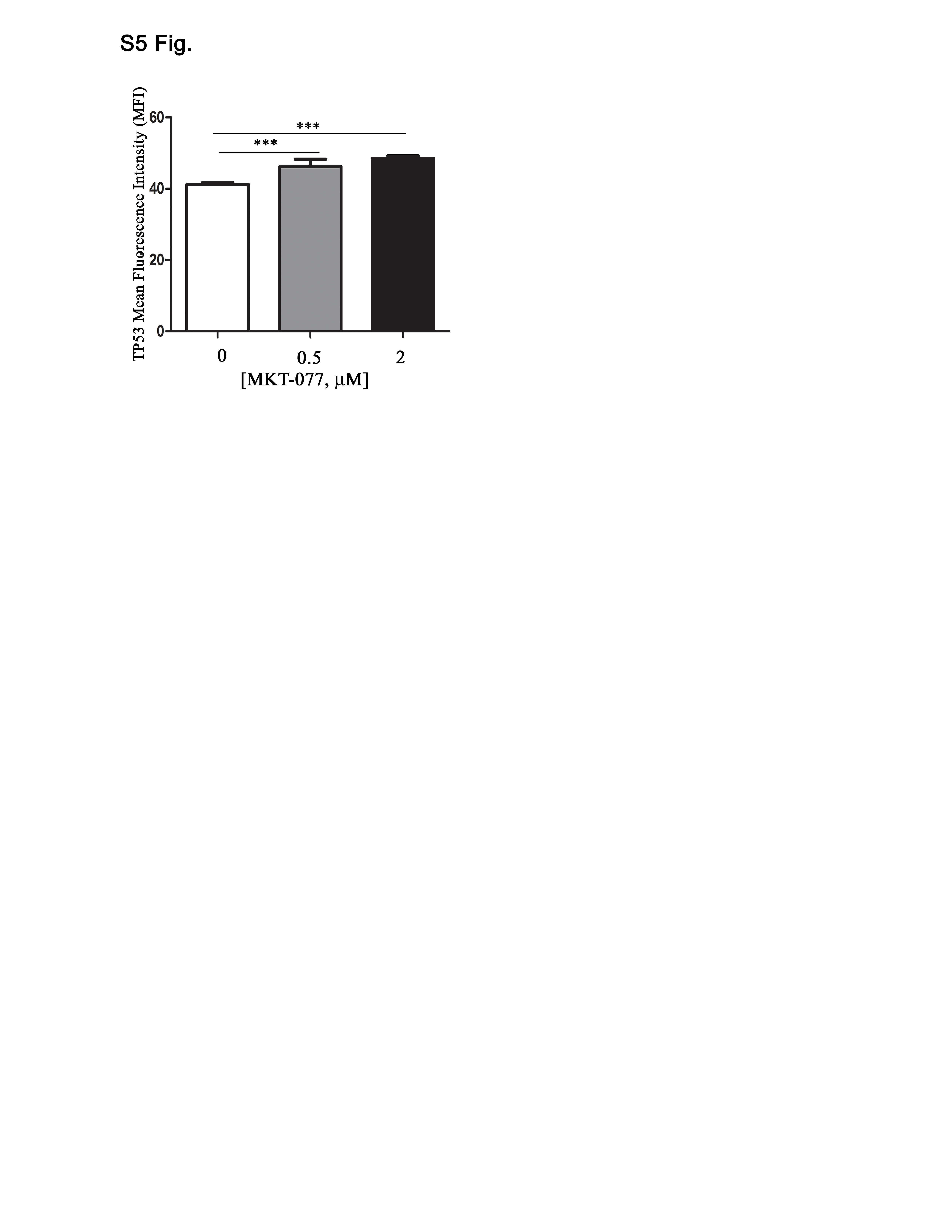

Supplement: S5 Fig — (A) Mean fluorescence intensity (MFI) of TP53 following treatment with various doses of MKT-077 (n = 3 technical replicates, representative of 2 independent experiments). ***p<0.001. (TIF) [file pone.0170470.s005.tif]

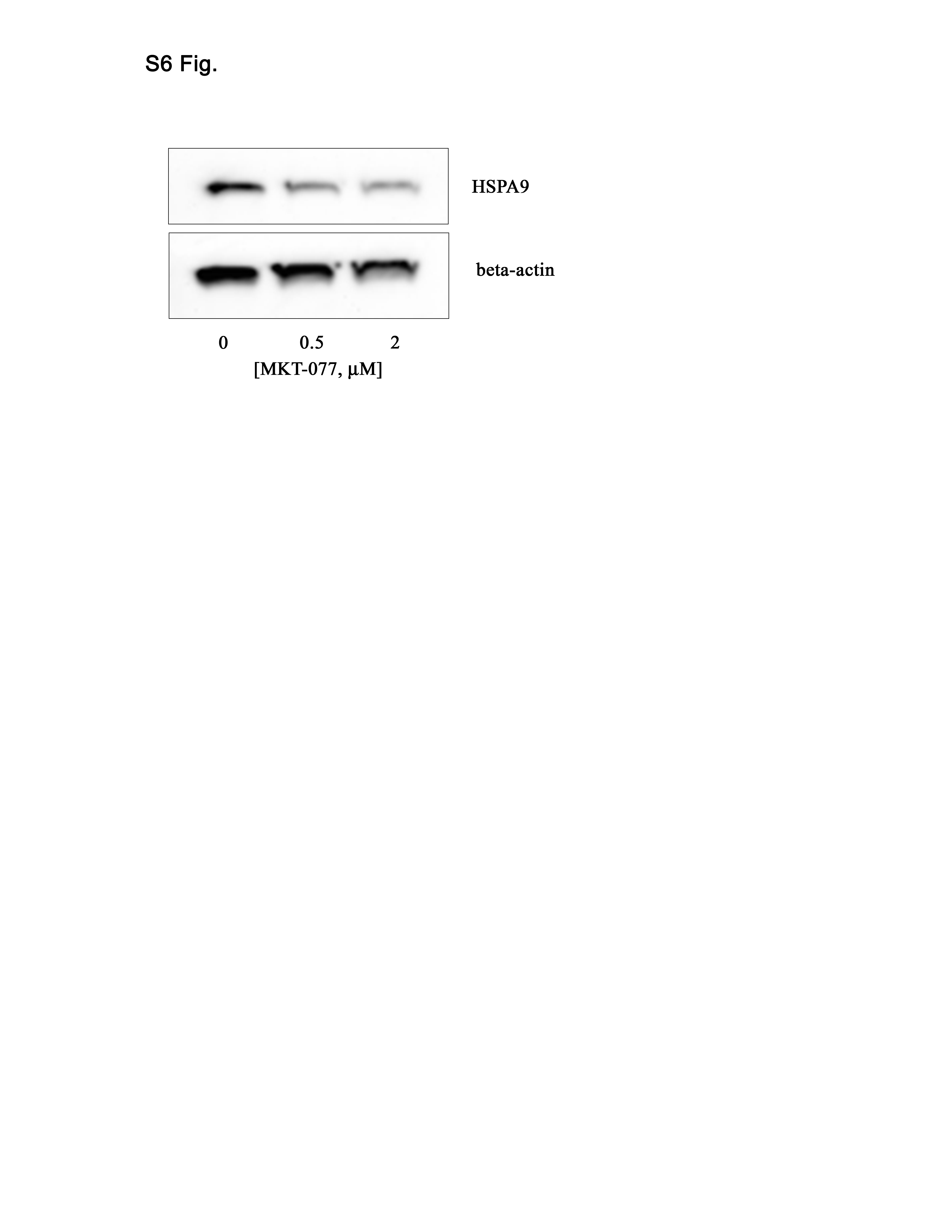

Supplement: S6 Fig — Bone marrow (BM) cells from a MDS patient with del(5q) were treated with various concentrations of MKT-077 for 4 days. Immunoblot of HSPA9 and beta-actin protein is shown. (TIF) [file pone.0170470.s006.tif]
