# Supplementary material for: Knockdown of HSPA9 induces TP53-dependent apoptosis in human hematopoietic progenitor cells
Source: PLoS One. 2017 Feb 8;12(2):e0170470. doi: 10.1371/journal.pone.0170470 (PMC5298293; doi:10.1371/journal.pone.0170470)
Supplement: S1 Table — (DOCX) [file pone.0170470.s007.docx]

**S1 Table. Short hairpin RNA sequences.**

| **shRNA** | **Vector** | **5’-sense** | | **Loop** | **Antisense-3’** |
| --- | --- | --- | --- | --- | --- |
| Luciferase | pLK0.1 | | CGCTGAGTACTTCGAAATGTC | CTCGAG | GACATTTCGAAGTACTCAGCG |
| GFP | pLK0.1 | | GCAAGCTGACCCTGAAGTTCA | CTCGAG | TGAACTTCAGGGTCAGCTTGC |
| HSPA9 (433) | pLK0.1 | | GCTGTCACCAACCCAAACAAT | CTCGAG | ATTGTTTGGGTTGGTGACAGC |
| HSPA9  (960) | pLK0.1 | | GCACATTGTGAAGGAGTTCAA | CTCGAG | TTGAACTCCTTCACAATGTGC |
| TP53#1  (309) | pLK0.1 | | TCAGACCTATGGAAACTACTT | CTCGAG | AAGTAGTTTCCATAGGTCTGA |
| TP53#2  (427) | pLK0.1 | | GTCCAGATGAAGCTCCCAGAA | CTCGAG | TTCTGGGAGCTTCATCTGGAC |
| TP53#3  (941) | pLK0.1 | | CACCATCCACTACAACTACAT | CTCGAG | ATGTAGTTGTAGTGGATGGTG |
| TP53#4  (1095) | pLK0.1 | | CGGCGCACAGAGGAAGAGAAT | CTCGAG | ATTCTCTTCCTCTGTGCGCCG |
| TP53#5  (1673) | pLK0.1 | | GAGGGATGTTTGGGAGATGTA | CTCGAG | TACATCTCCCAAACATCCCTC |
