# Supplementary material for: Knockdown of HSPA9 induces TP53-dependent apoptosis in human hematopoietic progenitor cells
Source: PLoS One. 2017 Feb 8;12(2):e0170470. doi: 10.1371/journal.pone.0170470 (PMC5298293; doi:10.1371/journal.pone.0170470)
Supplement: S2 Table — (DOCX) [file pone.0170470.s008.docx]

**S2 Table. Quantification of Western blot images by densitometry.**

**Figure 1A.**

|  | **shGFP** | **sh433** | **sh960** |
| --- | --- | --- | --- |
| **HSPA9/beta-actin** | 1.02 | 0.80 | 0.41 |
| **Normalized to shGFP** | 1 | 0.78 | 0.40 |
|  |  |  |  |
| **TP53/beta-actin** | 0.26 | 0.58 | 1.42 |
| **Normalized to shGFP** | 1 | 2.20 | 5.41 |

**Figure 2A.**

|  | **shGFP** | **sh433** | **sh960** |
| --- | --- | --- | --- |
| **p21/beta-actin** | 0.04 | 1.12 | 1.23 |
| **Normalized to shGFP** | 1 | 26.80 | 28.57 |
|  |  |  |  |
| **BAX/**  **beta-actin** | 0.59 | 1.17 | 2.00 |
| **Normalized to shGFP** | 1 | 1.98 | 3.39 |

**Figure 3B**

| **Cytoplasm** | | | | **Nuclear** | | | |
| --- | --- | --- | --- | --- | --- | --- | --- |
|  | **shGFP** | **sh433** | **sh960** |  | **shGFP** | **sh433** | **sh960** |
| **TP53/**  **beta-actin** | 1.02 | 0.14 | 0.16 | **TP53/**  **lamin B** | 0.09 | 0.22 | 1.17 |
| **Normalized to shGFP** | 1 | 0.14 | 0.16 | **Normalized to shGFP** | 1 | 2.43 | 13.01 |

**Figure 4A**

|  | **shGFP** | **#1** | **#2** | **#3** | **#4** | **#5** |
| --- | --- | --- | --- | --- | --- | --- |
| **TP53/**  **beta-actin** | 2.50 | 2.55 | 1.12 | 0.56 | 1 | 0.98 |
| **Normalized to shGFP** | 1 | 1.02 | 0.45 | 0.22 | 0.40 | 0.39 |

**S2 Table. Quantification of Western blot images by densitometry (continued)**

**Figure 5A.**

|  | **0 μM** | **0.5 μM** | **2 μM** |
| --- | --- | --- | --- |
| **HSPA9/beta-actin** | 1.39 | 1.31 | 0.26 |
| **Normalized to 0 μM** | 1 | 0.94 | 0.18 |
|  |  |  |  |
| **TP53/beta-actin** | 6.54 | 6.17 | 8.66 |
| **Normalized to 0 μM** | 1 | 0.94 | 1.32 |
|  |  |  |  |
| **BAX/beta-actin** | 0.28 | 0.37 | 1.30 |
| **Normalized to 0 μM** | 1 | 1.32 | 4.63 |
|  |  |  |  |
| **p21/beta-actin** | 0.10 | 1.00 | 1.08 |
| **Normalized to 0 μM** | 1 | 10.15 | 10.94 |

**Supplemental Figure 6.**

|  | **0 μM** | **0.5 μM** | **2 μM** |
| --- | --- | --- | --- |
| **HSPA9/beta-actin** | 0.78 | 0.38 | 0.38 |
| **Normalized to 0 μM** | 1 | 0.48 | 0.49 |
