# Supplementary material for: Knockdown of HSPA9 induces TP53-dependent apoptosis in human hematopoietic progenitor cells
Source: PLoS One. 2017 Feb 8;12(2):e0170470. doi: 10.1371/journal.pone.0170470 (PMC5298293; doi:10.1371/journal.pone.0170470)
Supplement: S3 Table — (DOCX) [file pone.0170470.s009.docx]

**S3 Table. TP53-induced and p21-inhibited gene lists used for GSEA analysis.**

TP53 induced and p21 inhibited gene lists used for GSEA analysis

|  | **TP53 induced genes** | **p21 inhibited genes** |
| --- | --- | --- |
| 1 | *CCNG2* | *ORC1L* |
| 2 | *PRODH* | *POLA1* |
| 3 | *PLK2* | *RRM1* |
| 4 | *TNFRSF10B* | *LIG1* |
| 5 | *XPC* | *MCM4* |
| 6 | *DDB2* | *MCM7* |
| 7 | *FGF2* | *CCNB1* |
| 8 | *CBLC* | *MAD2L1* |
| 9 | *RRAD* | *EXO1* |
| 10 | *COL4A1* | *RAD54L* |
| 11 | *ITGAM* | *RAD54L2* |
| 12 | *ASTN2* | *RAD54B* |
| 13 | *NAV3* | *ATRX* |
| 14 | *KCNMA1* | *CCNA2* |
| 15 | *SPANXB1* |  |
| 16 | *DENND2D* |  |
| 17 | *SHROOM3* |  |
| 18 | *DTWD1* |  |
| 19 | *GADD45A* |  |
| 20 | *BBC3* |  |
| 21 | *PCNA* |  |
| 22 | *BAX* |  |
| 23 | *CDKN1A* |  |
| 24 | *TP53AP1* |  |
| 25 | *RPS27L* |  |
| 26 | *PGPEP1* |  |
| 27 | *KIAA0564* |  |
| 28 | *MDM2* |  |
